# Comparison of the efficacy and tolerability of elobixibat plus sodium picosulfate with magnesium citrate and split-dose 2-L polyethylene glycol with ascorbic acid for bowel preparation before outpatient colonoscopy: a study protocol for the multicentre, randomised, controlled E-PLUS trial

**DOI:** 10.1186/s12876-024-03146-6

**Published:** 2024-02-03

**Authors:** Kinichi Hotta, Yosuke Otake, Daisuke Yamaguchi, Yuichi Shimodate, Norihiro Hanabata, Hiroaki Ikematsu, Yohei Yabuuchi, Yasushi Sano, Ryo Shimoda, Shinya Sugimoto, Mari Oba, Hiroyuki Takamaru, Kouichiro Kimura, Yoshihiro Kishida, Kazunori Takada, Sayo Ito, Kenichiro Imai, Kazuya Hosotani, Tatsuro Murano, Masayoshi Yamada, Kensuke Shinmura, Rio Takezawa, Michito Tomonaga, Yutaka Saito

**Affiliations:** 1 Division of Endoscopy, Shizuoka Cancer Center, 1007 Shimonagakubo, Nagaizumi-Cho, Sunto-Gun, Shizuoka, 411-8777 Japan; 2 Department of Gastroenterology, Subaru Health Insurance Society Ota Memorial Hospital, Ota, Japan; 3 Department of Gastroenterology, National Hospital Organization Ureshino Medical Center, Ureshino, Japan; 4 Department of Gastroenterology and Hepatology, Kurashiki Central Hospital, Kurashiki, Japan; 5 Department of Gastroenterology, Aomori Prefectural Central Hospital, Aomori, Japan; 6 Endoscopy Division, National Cancer Center Hospital East, Kashiwa, Japan; 7 Department of Gastroenterology, Kobe City Medical Center General Hospital, Kobe, Japan; 8 Gastrointestinal Center, Sano Hospital, Kobe, Japan; 9 Department of Internal Medicine, Division of Gastroenterology, Faculty of Medicine, Saga University, Saga, Japan; 10 Department of Gastroenterology, Japanese Red Cross Ise Hospital, Ise, Japan; 11 National Center of Neurology and Psychiatry, Tokyo, Japan; 12 Endoscopy Division, National Cancer Center Hospital, Tokyo, Japan

**Keywords:** Boston bowel preparation scale, Bowel preparation, Colonoscopy, Elobixibat, Polyethylene glycol, Sodium picosulfate magnesium citrate

## Abstract

**Background:**

Sodium picosulfate (SP)/magnesium citrate (MC) and polyethylene glycol (PEG) plus ascorbic acid are recommended by Western guidelines as laxative solutions for bowel preparation. Clinically, SP/MC has a slower post-dose defaecation response than PEG and is perceived as less cleansing; therefore, it is not currently used for major bowel cancer screening preparation. The standard formulation for bowel preparation is PEG; however, a large dose is required, and it has a distinctive flavour that is considered unpleasant. SP/MC requires a small dose and ensures fluid intake because it is administered in another beverage. Therefore, clinical trials have shown that SP/MC is superior to PEG in terms of acceptability. We aim to compare the novel bowel cleansing method (test group) comprising SP/MC with elobixibat hydrate and the standard bowel cleansing method comprising PEG plus ascorbic acid (standard group) for patients preparing for outpatient colonoscopy.

**Methods:**

This phase III, multicentre, single-blind, noninferiority, randomised, controlled, trial has not yet been completed. Patients aged 40–69 years will be included as participants. Patients with a history of abdominal or pelvic surgery, constipation, inflammatory bowel disease, or severe organ dysfunction will be excluded. The target number of research participants is 540 (standard group, 270 cases; test group, 270 cases). The primary endpoint is the degree of bowel cleansing (Boston Bowel Preparation Scale [BBPS] score ≥ 6). The secondary endpoints are patient acceptability, adverse events, polyp/adenoma detection rate, number of polyps/adenomas detected, degree of bowel cleansing according to the BBPS (BBPS score ≥ 8), degree of bowel cleansing according to the Aronchik scale, and bowel cleansing time.

**Discussion:**

This trial aims to develop a “patient-first” colon cleansing regimen without the risk of inadequate bowel preparation by using both elobixibat hydrate and SP/MC.

**Trial registration:**

Japan Registry of Clinical Trials (jRCT; no. s041210067; 9 September 2021; https://jrct.niph.go.jp/), protocol version 1.5 (May 1, 2023).

## Background

Colorectal cancer (CRC) is the second leading cause of death attributable to cancer in Japan; every year, it affects approximately 150,000 individuals and results in the death of approximately 50,000 individuals [1]. Increasing the screening rate using the faecal immunological test, which is currently being introduced as a population-based screening method, is considered the most effective method of reducing the mortality rate attributable to CRC. Total colonoscopy is a thorough examination; therefore, it is the first choice for patients who require further investigation. However, the current screening rate is less than 30% in Japan. Only approximately 70% of patients with positive faecal immunological test results undergo total colonoscopy [1]. One of the reasons for this is the acceptability of total colonoscopy.

Factors that contribute to the nonacceptance of colonoscopy include resistance to bowel cleansing, which is considered comparable to the pain associated with colonoscope insertion. Inadequate bowel preparation is known to reduce the quality of the examination because it affects the adenoma detection and caecal intubation rates, increases the incidence of post-colonoscopy CRC, shortens the interval between subsequent examinations, and increases costs [2].

Guidelines in Europe and the United States recommend dividing the doses of polyethylene glycol (PEG) as the most effective bowel cleansing method [2, 3].

Due to the recent increase in demand for colonoscopies, an increasing number of medical facilities have begun offering colonoscopies during morning hours. Therefore, the demand for split-dose methods for colonoscopies that involve a short time between the initial administration of the internal bowel preparation solution and the completion of bowel preparation is expected to increase in the future.

SP/MC has been approved in Japan and can be administered either on the day before the examination or as split doses. In Japan, a phase III, multicentre, randomised, controlled trial involving an SP/MC split-dose group, SP/MC 1-day group, and 2-L PEG same-day group was performed. The SP/MC split-dose resulted in the best colon preparation and was statistically noninferior to the 2-L PEG same-day dose. Additionally, SP/MC was significantly superior to PEG in terms of acceptability because of its ease of consumption, general impression, taste, and volume [4]. However, a meta-analysis of 25 randomised, controlled trials of SP/MC and PEG in Europe and the United States found no statistically significant difference in bowel cleanliness and a trend toward better cleanliness with PEG (relative risk [RR], 0.93; 95% confidence interval [CI], 0.86–1.01). Furthermore, the acceptability of SP/MC was significantly better (RR, 1.08; 95% CI, 1.04–1.13) [5].

The standard formulation of PEG requires a large dose of solution with a distinctive, unpleasant flavour; therefore, many individuals are resistant to its use. In contrast, SP/MC requires a small internal dose and ensures fluid intake because it is administered in another beverage. Clinical trials have shown that SP/MC is superior to PEG in terms of acceptability [6]. Therefore, if the cleaning speed and effectiveness of SP/MC are improved, and if physicians and patients feel that it is comparable to PEG, then it could become part of the mainstream bowel preparation method. This study aims to develop a new bowel preparation method including SP/MC and a combination of drugs that are expected to enhance the cleansing effect.

Recently, several drugs with completely different pharmacological actions have been developed to treat chronic constipation. Among them, elobixibat, which has pharmacological actions such as increasing the water content and transport capacity in the intestinal tract, is expected to enhance the cleansing effect when used in combination with existing bowel-cleansing agents.

## Methods/design

### Hypothesis

The main research hypothesis of this study is that the test regimen (split-dose SP/MC plus elobixibat) is not inferior to the standard regimen (split-dose of 2 L of PEG plus ascorbic acid) in terms of colon cleanliness. If the superiority of the test regimen compared to that of the standard regimen in terms of acceptability can be demonstrated, then the utility of the test regimen can be demonstrated.

### Objectives

A novel bowel cleansing method (test method) comprising SP/MC (Picoprep^Ⓡ^; Nippon Chemiphar Co., Ltd., Tokyo, Japan) and elobixibat hydrate (Goofice^Ⓡ^ Tablets; Mochida Pharmaceutical Co., Ltd., Tokyo, Japan) will be compared with a standard bowel cleansing method comprising PEG plus ascorbic acid (Moviprep^Ⓡ^; EA Pharma Co., Ltd., Tokyo, Japan) used by patients scheduled to undergo elective colonoscopy in an outpatient setting. A randomised comparison of these two methods will be performed.

### Design

**Fig. 1:**
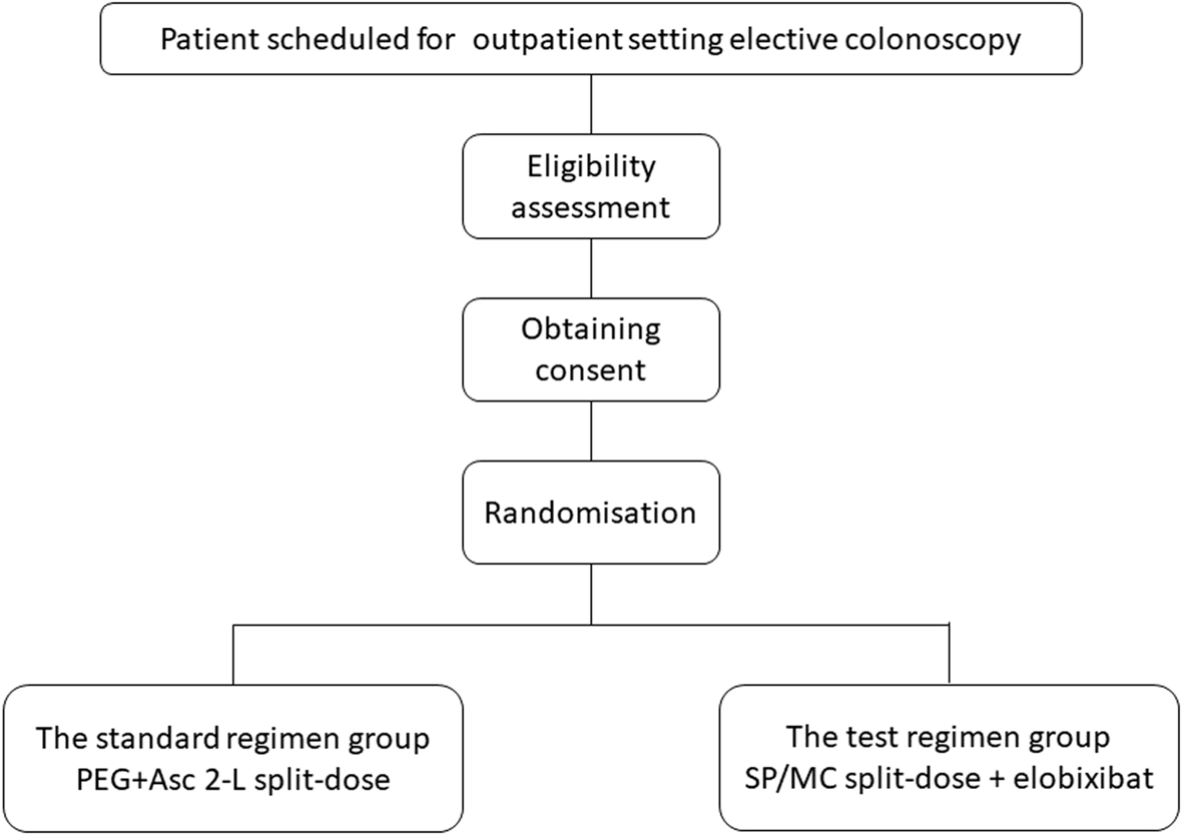
Diagram of the study design

This will be a phase III, noninferiority, multicentre, single-blind, randomised, controlled trial called the E-PLUS trial. A flowchart of the E-PLUS trial design is shown in Fig. 1.

### Randomisation

The principal investigator (or co-principal investigator) will confirm that patients who submit written consent are enrolled as research subjects have fulfilled all the inclusion criteria and none of the exclusion criteria. During this clinical research, patient information will be entered into the registration system (ANCHOR: Shizuoka Cancer Center Clinical Trial Support System; URL: https://www.anchor-scchr.net/). These patients will be registered as research subjects, and randomised by the minimization method. The four allocation adjustment factors will be facilities, age, sex, and inspection objectives.

### Blinding

Although it is difficult to blind patients who use medication for bowel preparation, endoscopists who will perform the test and assess the degree of colon cleanliness will be blinded to the allocation results. Therefore, this trial will have a single-blind design.

### Study population

Patients scheduled to undergo elective outpatient colonoscopy at a centre contributing to this study are eligible to participate in this trial.

### Inclusion criteria

The inclusion criteria are as follows:

1. Scheduled to undergo elective colonoscopy in an outpatient setting
2. Between 40 and 69 years of age at the time of providing consent
3. Freely provided written informed consent after receiving a full explanation of the study

### Exclusion criteria

The exclusion criteria are as follows:

1. Patients who have previously been enrolled in the present study
2. Allergy to the study drug
3. Eastern Cooperative Oncology Group (ECOG) Performance Status (PS) score of 2 or more
4. Diagnosis or suspicion of bowel obstruction or colonic stenosis (including obstruction caused by CRC).
5. Constipation (bowel movements less than three times per week or using medication for constipation)
6. Active gastroduodenal ulcer
7. Moderate or severe liver or renal dysfunction
8. Severe cardiac disease
9. Poorly controlled hypertension and diabetes mellitus
10. Use of antithrombotic drugs that are difficult to discontinue
11. Dysphagia or at risk for aspiration
12. History of abdominal or pelvic surgery (excluding endoscopic resection or haemorrhoid surgery).
13. Multiple colonic diverticula confirmed by colonoscopy or enema X-ray examination
14. Undergoing treatment for inflammatory bowel disease or colonic polyposis
15. Undergoing treatment for irritable bowel syndrome or functional dyspepsia
16. History of ischemic colitis
17. Pregnancy, possible pregnancy, within 28 days postpartum, or lactating
18. Psychiatric disorders or symptoms that interfere with daily life and render it difficult to participate in the study
19. Regular use of anticholinergic drugs, lithium, intestinal peristaltic drugs, opioids, or iron preparations
20. Body mass index ≤ 17 or body mass index ≥ 35
21. The following abnormal laboratory blood test results within 28 days of registration and eligibility confirmation:
  ① Aspartate aminotransferase ≥ 120 U/L
  ② Alanine aminotransferase ≥ 120 U/L
  ③ Serum creatinine ≥ 1.2 mg/dL
  ④ Serum sodium < 130 mEq/L
  ⑤Serum potassium < 3.0 mEq/L

### Participating hospitals

The E-PLUS trial will be conducted in Japan at seven community-based hospitals, three cancer centre hospitals, and one university hospital. The following hospitals will be participating in the trial: Shizuoka Cancer Center; National Cancer Center Hospital; Subaru Health Insurance Society Ota Memorial Hospital; National Hospital Organization Ureshino Medical Center; Kurashiki Central Hospital; Aomori Prefectural Central Hospital; National Cancer Center Hospital East; Kobe City Medical Center General Hospital; Sano Hospital; Saga University Hospital; and Japanese Red Cross Ise Hospital.

### Trial intervention

The enrolled patients will be scheduled to undergo colonoscopy between 2 and 90 days from the date of registration. The patients will be allocated to either the standard group or test group. They will consume only low-residue meal packets during the day before the colonoscopy. Laxative use and the internal use of intestinal peristalsis-promoting drugs in combination with pre-treatment drugs are not allowed. Patients using oral antithrombotic drugs should take measures such as medication withdrawal in accordance with the guidelines for gastrointestinal endoscopy practice for patients using antithrombotic drugs and supplement 2017 regarding anticoagulants, including direct oral anticoagulants, published by the Japanese Society of Gastrointestinal Endoscopy. Heparin bridges will not be performed because of the high incidence of posterior haemorrhages. A protocol including the examination of the results of ingesting a low-residue meal pack the day before colonoscopy and the administration of bowel-cleansing agents and additional fluids will be specified for each group and performed at the end of the colonoscopy. Endoscopic resection performed during the protocol examination will be included in the protocol examination, but any additional treatment to be performed at a later date is not specified. The 30-day follow-up period comprises the end of the protocol.

### Standard group

After dinner on the day before the colonoscopy, between 6:00 pm and 7:00 pm, the patient will consume 1 L of PEG plus ascorbic acid and 0.5 L of fluid (water or tea) over the course of at least 1 h. On the day of the colonoscopy, the patient will fast and consume 1 L of PEG plus ascorbic acid and 0.5 L of fluid (water or tea) over the course of at least 1 h between 4 and 5 h before the scheduled start of the examination.

### Test group

Two 5-mg elobixibat hydrate tablets (total of 10 mg) will be administered before breakfast on the day before the colonoscopy. After dinner on the day before the colonoscopy, one packet of SP/MC dissolved in 150 mL of water between 6:00 pm and 7:00 pm will be consumed. Then, four doses of 250 mL of a clear beverage (water, tea, sports drink, clear juice, or clear carbonated drink) will be consumed at least 1 h apart. Patients will fast 4–5 h before the scheduled start of the colonoscopy. Additionally, they will consume one packet of SP/MC dissolved in 150 mL of water. Then, they will consume four more 250-mL doses of a clear beverage (water, tea, sports drink, clear juice, or clear carbonated drink) at least 1 h after consuming the SP/MC dose.

### Colonoscopy procedures

1. The defaecation status and subjective symptoms will be evaluated after the examination.
2. Participants will be asked to complete a questionnaire before the colonoscopy.
3. Blood samples will be collected on the same day to detect electrolyte, renal and liver function abnormalities due to bowel preparation. Blood samples will be collected after the end of the examination if they cannot be collected before the colonoscopy.
4. All tests conducted during this study will be conducted as outpatient tests.
5. A deviation from the protocol will be considered if an inpatient examination is required.
6. Hospital admission after the completion of the colonoscopy will not be considered a deviation from the protocol.
7. The level of the examiner will be intermediate or higher (experience with at least 500 colonoscopies).
8. The examining physician will be blinded to the allocation group. The physician who prescribes the pre-treatment drug and the examining physician will be different. If the same physician performs both procedures, then those results will be excluded from the per-protocol analysis.
9. The procedure will be performed using a general colonoscope. The manufacturer and model of the scope used will not be specified.
10. Intravenous administration of the antispasmodic butyl scopolamine or glucagon will be recommended.
11. Intravenous opioid analgesics will be recommended.
12. Intravenous administration of benzodiazepine sedatives will be acceptable.
13. This study will assume total resection of tumorous lesions detected during the protocol examination and will document the endoscopic resection method and pathological results.
14. Serrated polyps will be, in principle, resected, except for those smaller than 5 mm on the left-sided colon and the rectum. However, priority will be given to resecting adenomatous polyps. Serrated adenomas and adenomas usually will be treated similarly.
15. The location, gross type, tumour diameter, and endoscopic diagnosis (serrated polyps, adenomas, early CRC, advanced CRC, and neuroendocrine tumours) of neoplastic lesions will be recorded.
16. The method of resection and the medical equipment will not be specified.
17. Pathological results will indicate the diagnosis and, in cases of cancer, the depth of invasion.
18. Lesions planned for endoscopic resection or surgery at a later date will be described in terms of the endoscopic diagnosis (e.g., serrated polyps, adenomas, early CRC, advanced CRC, and neuroendocrine tumours) and planned treatment (e.g., endoscopic mucosal resection, endoscopic submucosal dissection, and surgery).
19. The cecal intubation time and withdrawal time will be recorded.
20. After completion of the colonoscopy, the examining physician will record the bowel cleansing status according to the Boston Bowel Preparation Scale (BBPS) and Aronchik scale.
21. If a colonoscope is inserted and the total colonoscopy is deemed impossible to perform because of poor preparation, then it will be permissible to perform additional preparation before attempting the colonoscopy again on the same day after the BBPS and Aronchik scale scores have been assessed. If re-examination is not feasible on the same day, then it will be considered a deviation from the protocol inspection provisions and routine performance of the colonoscopy.

### Questionnaire for patients

Patients will be asked to complete a questionnaire that includes the following:

Presence and severity of the following subjective symptoms (assessed using a 5-point scale) before the colonoscopy: abdominal pain, abdominal distension, nausea, vomiting, headache, dizziness, and general fatigue.

The acceptability of the bowel preparation regimen will be determined using a 5-point scale:

1. Was the bowel preparation medication easy to use?
2. Were all bowel preparation medicines administered internally?
3. What is your general impression of the bowel preparation?
4. How did the bowel preparation medicine taste?
5. Did you have a bowel movement promptly after using the bowel preparation medication?
6. Did your stools become clear after using the bowel preparation medication?
7. Did you wake during the night to defecate?
8. Were you able to sleep the day before the examination?
9. Did you have to stop to use a toilet on the way to the examination facility?
10. Do you want to use the same bowel preparation medication for your next colonoscopy?

Presence and severity of subjective symptoms during colonoscopy (assessed using a 5-point scale): insertion pain, abdominal distension, nausea, and vomiting.

Presence and degree of subjective symptoms after colonoscopy (assessed using 5-point scale): abdominal pain, abdominal distension, nausea and vomiting, constipation, diarrhoea, bloody stools, fever, and general malaise.

### Outcomes

#### Primary endpoint

The primary endpoint is the degree of bowel cleansing (BBPS score ≥ 6). Each segment should receive a score of at least 2 points—thus yielding a total of at least 6 points.

#### Secondary endpoints

The secondary endpoints are as follows:

1. Patient acceptability (questionnaire)
2. Adverse event
3. Polyp/adenoma detection rate and number of polyps/adenomas detected
4. Degree of bowel cleansing (percentage of BBPS scores > 8)
5. Degree of bowel cleansing (percentage of Aronchik scale scores < 1)
6. Bowel cleansing time (time from the start of bowel preparation until the time when the patient is ready for the examination)

#### Sample size calculation

The target number of research participants was 540 (standard group, 270 cases; test group, 270 cases). Based on previous studies, the percentage of the standard group with a bowel cleanliness score ≥ 6 should be approximately 90% [2]. The percentage of the test group with this score is expected to be similar or slightly lower, at 88–90% [2]. The standard cleaning method prior to the current standard bowel cleansing method was 4 L of PEG the day before the colonoscopy; this regimen was considered inferior (inferiority margin, 15%) in terms of good cleaning scores when compared to the split-dose regimen comprising 2 L of PEG plus ascorbic acid. Therefore, a margin of noninferiority of 10% was deemed appropriate. Based on a one-sided alpha error of 2.5%, a beta error of 20%, and a noninferiority margin of 10% for the standard and test groups, the required number of subjects in each group is 255. However, considering a 5% assessment failure rate, the target study population will be 270 subjects in each group. The Farrington–Manning method was used with SAS (version 9.4; SAS Institute, Cary, NC, USA) to calculate the required number of participants [7].

### Duration of clinical trial

Based on preliminary interviews with participating centres, approximately 35 participants per month are expected to be enrolled; therefore, approximately 16 months is estimated to be required to enrol 540 participants. Considering the monthly variations in enrolment numbers, the initial enrolment period was 18 months (1 year and 6 months). However, the number of enrolled patients did not reach the target within that time; therefore, the registration period was extended to 30 months as of 22 November 2022.

Total study period: 3 years and 10 months (1 October 2021 to 31 July 2025).

Registration period: 2 years and 6 months (1 October 2021 to 31 March 2024).

Follow-up period: 30 days from the endoscopy date of each participant.

Analysis period: 1 year from the end of the follow-up period.

### Ethics

All investigators involved in this clinical study will conduct it in accordance with the Declaration of Helsinki (translated by the Japan Medical Association), the Clinical Research Act (act no. 16 of 2009), the Enforcement Regulations for the Clinical Research Act (Ministry of Health, Labour and Welfare Ordinance [MHLW] no. 17 of 2008), and related notices from the Japanese government. Prior to the start of this clinical study, the principal investigator will obtain approval from the administrator of the medical institution to conduct the clinical study after obtaining the opinion of the certified review board and submit an implementation plan to the MHLW.

The principal investigator(s) will explain the research in a manner that is easy to understand to the potential research subject(s) using an ‘explanation document’ approved by the certified review board and obtain written, voluntary consent to participate in the research. When obtaining consent, the potential research subject should be given sufficient time and opportunity to ask questions and have them answered fully to enable the potential research subject to decide whether to participate in the study.

### Analysis population

For the purpose of this study, the population to be analysed is defined as follows. In principle, the full analysis set will be used as the analysis population for the efficacy endpoints, and the safety analysis set will be used as the analysis population for the safety endpoints. The per-protocol set will be used as the analysis population if necessary. As this study is positioned as a validation study, the analysis will be conducted using a modified intention-to-treat analysis during which patients will be classified into groups according to their allocation and not according to the actual bowel-cleansing method used.

### Full analysis set

The full analysis set will include all participants enrolled in the clinical study who are randomised to the study but excluded for the following reasons:

1. Violation of the eligibility/exclusion criteria
2. No available data after randomisation (e.g., withdrew consent, no future evaluations)
3. Withdrawal of consent, including consent for data use
4. Failure to participate in the protocol testing
5. Failure to undergo colonoscopy for reasons unrelated to the pre-treatment

### Per protocol set

The per protocol set is defined as all participants in the full analysis set except for the following:

1. Those who did not receive the assigned pre-treatment
2. Those who were not performed blinded evaluation because the physician prescribing the pre-treatment drug and the examining physician were the same physician

### Safety analysis set

The study population will consist of all enrolled patients for whom protocol testing is initiated.

### Data collection

An electronic data capture system will be used to collect the data necessary for this clinical study. The person responsible for preparing the case report form will promptly enter the necessary data into the electronic data capture system and confirm that the information regarding individual research subjects has been recorded correctly. Authentication and access rights to the electronic data capture system will be secured using identifiers and passwords, and encrypted communication will be used for data transmission.

### Statistical analysis plan

Participant characteristics are summarized descriptively. The number of observations, mean, standard deviation, minimum, median, maximum and interquartile range of continuous variables and frequency distributions of categorical variables are calculated.

The proportion of patients with a bowel cleanliness score ≥ 6 is calculated for each group. The difference between groups and 95% confidence interval is estimated using Miettinen-Nurminen method. Noninferiority will be declared when the lower limit of the confidence interval is above the margin. For secondary purpose, we plan to conduct the adjusted analysis using regression models and subgroup analysis restricted to the participants who answer “take more than half” or “take all of the medication.”

Secondary endpoints will bel summarized appropriately and compared between the groups using nonparametric tests. Multiplicity adjustment will not perform for the secondary endpoints.

Frequency distributions of the grade of adverse events will be summarized for each group. Group comparison will be performed using the Fisher’s exact test, if necessary.

### Trial registration

After notifying the MHLW of the plan for conducting this clinical research, the principal investigator will register the plan for conducting this clinical research in the Japan Registry of Clinical Trials (jRCT; no. s041210067; 9 September 2021; https://jrct.niph.go.jp/), which is a system established by the MHLW for the submission and publication of clinical research, and make it public. The registered information will be updated according to the progress of the research.

## Discussion

This study aims to evaluate the degree of bowel cleansing and acceptability of a new regimen comprising split-dose SP/MC plus elobixibat hydrate and compare it with the standard regimen comprising split-dose PEG plus ascorbic acid by performing a randomised comparison. The primary endpoint is the degree of bowel cleansing according to the BBPS score of the noninferior design. The secondary endpoint is acceptability, which will be determined using a questionnaire regarding the superiority of the design. If the test regimen is not inferior to the standard regimen according to the primary endpoint, and if the superiority of the test regimen compared to that of the standard regimen in terms of acceptability can be demonstrated, then the utility of the test regimen can be demonstrated.

PEG is the most commonly recommended solution for bowel preparation according to the guidelines of Western countries because of its colon-cleansing effectiveness [2, 3]. Recently, a split dose has been recommended not only for colon cleansing but also for improving the adenoma detection rate [8]. However, PEG has some problems in terms of patient acceptability, such as the need for a large dose and its flavour.

In Japan, SP/MC has been administered as split doses for several years. However, the post-dose defaecation response of SP/MC is slower than that of PEG, and it is perceived as having a lesser cleansing effect; therefore, it is not currently used for major bowel cancer screening preparation. We believe that the good acceptability of SP/MC compared to that of PEG is worth noting. Therefore, a new regimen including elobixibat hydrate, which does not influence acceptability and safety, has been proposed. According to the current Western guidelines, the use of adjunctive agents for bowel cleansing is not recommended because of the lack of evidence regarding the effectiveness. Lubiprostone and elobixibat hydrate have been used as adjunctive agents for bowel preparation during randomised trials. However, those trials did not demonstrate robust effectiveness. Elobixibat is currently indicated by insurance as a treatment for chronic constipation, but not as a colonoscopy preparation agent. The results of a domestic clinical trial of chronic constipation showed that elobixibat produced spontaneous defaecation among 85.5% of patients; this rate was significantly higher than that of the placebo (41.3%). During the same study, the median time to onset of the first spontaneous bowel movement was 5.2 h, which was significantly shorter than that for placebo (25.5 h) [9]. These results suggest that elobixibat could be expected to have an immediate effect, and that it could be an effective pre-treatment adjunctive agent for those scheduled to undergo colonoscopy.

In Japan, a previous multicentre, randomised, controlled trial of elobixibat as a pre-treatment for colonoscopy demonstrated noninferior bowel cleanliness with the test method (PEG plus ascorbic acid plus elobixibat group: group A) and the standard method (PEG plus ascorbic acid plus picosulfate group: group B). Adequate bowel preparation rate as a primary endpoint was 95.0% vs 99.0% in group A and B, and noninferiority was proved. Furthermore, sleep disturbance was 10.2% and 22.7% (*P* = 0.02) in group A and B [10]. Moreover, cost of two tablets of elobixibat is very low at 178.4 JPY (0.96 GBP). Therefore, 10 mg of elobixibat before breakfast on the day before the colonoscopy has been selected as the concomitant drug for the test regimen.

The main target population of this study will comprise those with the following indications for outpatient colonoscopy: 40–69 years of age; positive faecal occult blood test results; colorectal lesions requiring close examination; surveillance required after polypectomy; and bloody stools or abnormal bowel movements experienced by symptomatic and asymptomatic patients. The exclusion criterion will be the distinct risk of inappropriate bowel preparation. To be eligible for study inclusion, the entire colon must be preserved so that the results will be widely transferable to clinical practice. Those who have undergone colorectal resection will be excluded. Abdominal and pelvic surgeries other than colorectal resection may have various effects on adhesions and intestinal peristalsis, and their impact on the assessment of the primary endpoint is unpredictable; therefore, patients who have undergone such surgeries will be excluded. Patients with conditions such as inflammatory bowel disease, irritable bowel syndrome, and chronic constipation will be excluded because of their impact on pre-treatment outcomes. Therefore, patients scheduled for a wait-list outpatient examination who can be adequately informed in advance, except those who fulfil the aforementioned exclusion criteria, will be included. Eligible patients will be aged at least 40 years, and they will remain the same age during the study period. Patients will not be older than 69 years because older individuals are generally at increased risk for adverse events attributable to bowel cleansing.

Several randomised, controlled trials and meta-analyses have shown that cleanliness is good when the standard regimen is used [11]. However, the acceptability of the standard regimen is not favourable because of its flavour and high oral dose. SP/MC has been well-accepted by patients during several randomised, controlled trials because of its low oral dose, and because the patients can choose from a variety of clear beverages [5]. SP/MC has been reported to be inferior to standard regimens in terms of cleanliness; however, the concomitant drug elobixibat hydrate may improve cleanliness. At present, this mechanism remains unknown. Patients participating in the study will be expected to exhibit better bowel cleansing than those encountered in standard practice because they will be fully briefed in advance regarding the colonoscopy pre-treatment; as a result, they may undergo a more accurate colonoscopy.

Adverse reactions to the drugs used in this study may occur. Serious adverse reactions associated with bowel cleansing include anaphylaxis, bowel perforation, bowel obstruction, incarcerated inguinal hernia, hyponatraemia, and ischaemic colitis; however, the risks of these reactions are considered similar to those encountered in usual practice. The risks of these adverse events associated with colonoscopy and lesion treatment are also considered comparable to those encountered in usual practice.

During this study, the primary endpoint will be evaluated based on the percentage of those with adequate bowel preparation (BBPS score ≥ 6); therefore, a secondary endpoint is excellent bowel preparation (BBPS score ≥ 8).

The limitations of this study include the single-blind design, difficulty evaluating the independent effectiveness of elobixibat hydrate because of the study design, and difficulty excluding selection bias during the evaluation of the adenoma detection rate as a secondary endpoint.

In conclusion, this trial aims to develop a “patient-first” regimen for colon cleansing among patients who are not at risk for inadequate bowel preparation.

## Data Availability

Data sharing is not applicable to this article as no datasets were generated or analysed during the current study.

## Abbreviations

SP/MC: Sodium picosulfate magnesium citrate
PEG: Polyethylene glycol
Asc: Ascorbic acid
BBPS: Boston Bowel Preparation Scale
jRCT: Japan Registry of Clinical Trials
RR: Relative risk
CI: Confidence interval
PS: Performance status
ECOG: Eastern Cooperative Oncology Group
BMI: Body mass index
AST: Aspartate aminotransferase
ALT: Alanine aminotransferase
MHLW: Ministry of Health, Labour and Welfare Ordinance
CRB: The Certificated Review Board
FAS: The Full Analysis Set
SAS: The Safety Analysis Set
PPS: The Per Protocol Set
ITT: Intention-to-treat
EDC: The Electronic Data Capture
ADR: Adenoma detection rate
